# Evaluation of foot self-care status and foot screening problems in patients with diabetes in Iran: a national multicenter study

**DOI:** 10.1186/s12902-023-01401-7

**Published:** 2023-08-21

**Authors:** Mohammad Reza Amini, Mahnaz Sanjari, Mohamad Reza Mohajeri Tehrani, Ensieh Nasli, Leila Yazdanpanah, Zohreh Mousavi, Farzad Forghan, Neda Valizadeh, Mohammad Hossein Gozashti, Mohammad Afkhami-Ardekani, Mansour Siavash, Katayoun Vahdat, Abbas Shamsi, Donya Sadeghi, Bagher Larijani, Neda Mehrdad, Maryam Aalaa

**Affiliations:** 1 Diabetes Research Center, Endocrinology and Metabolism Clinical Sciences Institute, Tehran University of Medical Sciences, Tehran, Iran; 2 Osteoporosis Research Center, Endocrinology and Metabolism Clinical Sciences Institute, Tehran University of Medical Sciences, Tehran, Iran; 3 Endocrinology and Metabolism Research Center, Endocrinology and Metabolism Clinical Sciences Institute, Tehran University of Medical Sciences, Tehran, Iran; 4 Diabetes Research Center, Health Research Institute, Ahvaz Jundishapur University of Medical Sciences, Ahvaz, Iran; 5 Metabolic Syndrome Research Center, Mashhad University of Medical Sciences, Mashhad, Iran; 6 Gilan University of Medical Sciences, Gilan, Iran; 7 Maternal and Childhood Obesity Research Center, Urmia University of Medical Sciences, Urmia, Iran; 8 Endocrine and Metabolism Research Center, Institute of Basic and Clinical Physiology Sciences, Kerman University of Medical Sciences, Kerman, Iran; 9 Shahid Sadoughi University of Medical Sciences, Yazd, Iran; 10 Isfahan Endocrine and Metabolism Research Center, Isfahan University of Medical Sciences, Isfahan, Iran; 11 The Persian Gulf Tropical Medicine Research Center, The Persian Gulf Biomedical Sciences Research Institute, Bushehr University of Medical Sciences, Bushehr, Iran; 12 Department of Clinical Psychology, Khomein Branch, Islamic Azad University, Khomein, Iran; 13 Faculty of Nursing and Midwifery, Tehran University of Medical Sciences, Tehran, Iran; 14 Elderly Health Research Center, Endocrinology and Metabolism Population Sciences Institute, Tehran University of Medical Sciences, Tehran, Iran; 15 Department of e-Learning in Medical Education, Center of Excellence for e-Learning in Medical Education, School of Medicine, Tehran University of Medical Sciences, Tehran, Iran; 16 Evidence Based Medicine Research Center, Endocrinology and Metabolism Clinical Sciences Institute, Tehran University of medical Sciences, Tehran, Iran

**Keywords:** Diabetic Foot, Screening, Peripheral neuropathy, Self-care, Iran

## Abstract

**Background and purpose:**

The lack of timely foot care among individuals with diabetics often lead to ulceration followed by infection and amputation. This study aimed to evaluate the foot self-care status and foot screening practices among patients with type 2 diabetes in various cities across Iran.

**Methods:**

The cross-sectional descriptive study was performed on patients with type 2 diabetes in 10 main cities of Iran. The information about demographic and lifestyle factors, diabetes history, and diabetic foot self-care (DFSQ) was assessed. Additionally, the neurological and vascular condition of the foot were screened by Inlow’s 60-Second Screen.

**Results:**

The study included 1094 diabetic patients with, with a majority being female (64.8%) and married (92.5%). The average age of the participants was 57.6 ± 10.21 (mean ± SD), and the mean duration of diabetes was 11.56 ± 7.41 years. Based on Inlow’s 60-Second Screen criteria, 58% of the patients should undergo yearly foot ulcer screening, 47% exhibited peripheral neuropathy, and 37% were found to have inappropriate footwear.

**Conclusion:**

The high prevalence of peripheral neuropathy observed in approximately half of the participants across different regions of Iran underscores the importance of continuous patient education regarding foot care and appropriate footwear. Furthermore, regular foot ulcer screenings, following the recommended intervals outlined in Inlow’s screening protocol, should be implemented to effectively manage diabetic foot complications.

## Introduction

Diabetic Foot Ulcer (DFU) stands as one of the most prevalent complications of Diabetes Mellitus (DM) [1]. Globally, DFU prevalence reaches 6.3% [2], while in Iran, it ranges from 3 to 10.6% [3, 4]. Notably, estimations indicate that approximately 25% of individuals with DM will face the risk of developing DFUs during their lifetime [5].

DFUs, primarily associated with neuropathy and ischemia, constitute the leading cause of lower extremity amputation [6]. Moreover, DFUs and amputation are significant contributors to diabetes-related hospital admissions [7]. Given the prevalence of comorbidities and socioeconomic factors among these patients, the outcomes of lower extremity amputations carry significant implications [8]. Early postoperative mortality rates in this context range from 4 to 22%, highlighting the critical need for identifying risk factors and giving utmost attention to this matter [9].

Despite the considerable burden imposed by DFUs on patients, their families, and society, it is worth noting that most foot ulcers and resulting amputations are preventable. High-risk individuals can be identified through a history of previous ulceration or amputation and a comprehensive clinical examination that encompasses factors such as impaired monofilament sensation and vibration perception, absent Achilles tendon reflex, callus, foot deformities, inappropriate footwear, and absent pedal pulse. Among the numerous interventions aimed at preventing foot ulceration and its consequences, early recognition of high-risk patients and prompt referral to appropriate multidisciplinary teams stand as crucial steps [10, 11]. In this regard, prevention can be viewed from two primary dimensions.

The first dimension revolves around the foot screening of patients with diabetes. Regularly scheduled screening foot exams using validated and reliable tools can effectively identify individuals at risk of developing DFUs [12, 13]. Several diabetes associations and clinical practice guidelines in the field of DFU prevention and management recommend routine foot examinations for patients with diabetes at regular interval [14, 15]. The International Working Group on the Diabetic Foot (IWGDF) specifically advocates for an annual foot examination for all patients with diabetes, with more frequent screenings for those at a higher risk. Early identification of high-risk feet remains a top priority and an essential step in DFU prevention, given the clinical and economic burden associated with diabetic foot complications [16].

The second preventing dimension emphasizes the critical importance of foot self-care as a well-known strategy for preventing DFUs and their complications [17, 18]. Adequate knowledge and implementation of effective foot self-care practices by patients play a pivotal role in reducing the incidence of DFUs [11, 19]. However, further studies are required to assess the foot care knowledge and practice of patients with diabetes [19, 20].

In line with the revised recommendation, annual education on foot care and regular inspections are advised for all patients with diabetes, with increased frequency every three months for those with a history of ulceration, foot deformities, or neuropathy. Referral is recommended when foot ulceration fails to heal within two weeks or when the ulcer is deep [11, 14].

Given that a lack of timely screening and appropriate foot care for patients with diabetes can lead to ulceration and subsequent amputation, this study aims to explore the foot self-care status and the challenges surrounding foot screening for DFU risk among patients with DM in Iran. The diverse cultural and ethnic differences across different regions of Iran have prompted the selection of various provinces from the south, north, east, west, and central regions of the country to conduct this study.

## Methods

### Objectives

The objective of this study was to assess the foot self-care status and foot screening practices for the risk of developing DFUs among patients with DM in Iran.

### Study design

The cross-sectional descriptive study was performed in 10 provincial capital cities of Iran, including Rasht, Ahvaz, Tehran (2 centers), Mashhad, Urmia, Khomein, Isfahan, Bushehr, Yazd, and Kerman to evaluate the foot self-care status and screening of patients with diabetes in Iran.

### Study population

The study population consisted of all patients with type 2 diabetes referred to diabetes care at one of the following health centers in the ten mentioned cities as nodes of the diabetic foot network in Iran. Inclusion criteria were diagnosis of type 2 diabetes and the absence of active wounds or infections in the legs. A convenience sampling method was employed, and a total of 1094 patients were enrolled in the study over a 12-month period, from April 2019 to March 2020.

### Variables

In this study, the information about demographic and lifestyle factors (age, marital, educational, and social economic status), general health status (health insurance, life satisfaction, self-rated health), and medical history (DM duration, anti-diabetes medication, family history of DM, DM complication) was collected. Moreover, the Diabetic Foot Self-Care Questionnaire (DFSQ), composed of 16 items in three dimensions of “personal self-care,” “podiatric care,” and “footwear and socks,” was completed. DFSQ has been tested for validity and reliability [21, 22].

The 60 s Diabetic Foot Screening Tool used to identify patients at high risk of foot ulcers is known as the ‘Inlow’s 60-Second Screen’[23]. Inlow’s 60-Second Screen consists of three general categories, including look (20 seconds), touch (10 seconds), and assess (30 seconds). Skin, nails, deformity, and footwear are the components of the look category (4 items). Foot temperature (cold & hot) and range of motion are examined in the touch category (3 items). Sensation (Monofilament Testing and asking four questions), pedal pulses, dependent rubor, and erythema are assessed in the third category (5 items) [24]. This tool contains 12 items with scoring ranges from 0 to 25. The score ranges from 0 to 6 need yearly screening; ranges from 7 to 12 require every 6-month screening; ranges from 13 to 19 need every 3-month screening; and ranges from 20 to 25 should have screening every 1–3 month. The validity and reliability of this screening tool for diabetic foot risk stratification were confirmed in several studies [24, 25].

Inlow’s 60-Second Screen consists of three general categories, including look (20 s), touch (10 s), and assess (30 s). Skin, nails, deformity, and footwear are the components of the look category. Foot temperature (cold-hot) and range of motion are examined in the touch category. Sensation (Monofilament Testing and asking four questions), pedal pulses, dependent rubor, and erythema are assessed in the third category [24].

### Data gathering

Patients referred to the diabetes clinic were selected based on the inclusion criteria. First, the aim of the study was explained to the patient; then, the patient signed the informed consent form.

After that, a nurse responsible for data gathering completed the questionnaires and examinations.

The average time allocated to each patient to complete the questionnaire and perform the examination was about 20 min. Finally, the patient is taught based on the findings of examinations to meet ethical standards.

### Statistical analysis

The Kolmogorov-Smirnov test was used to measure the normality of the statistical sample. Additionally, the descriptive statistic, analysis of variance, and independent t-test were used to assess the associations between Inlow’s 60-Second Screen tool parameters, DF Self Care, and other determinants. A P value of < 0.05 was considered significant. The SPSS software version 16 was used for analysis.

## Results

A national multicenter study was conducted in Iran, involving a total of 1094 patients with diabetes. The study included participants from 10 provincial capital cities, namely Rasht, Ahvaz, Tehran (2 centers), Mashhad, Urmia, Khomein, Isfahan, Bushehr, Yazd, and Kerman. The aim of the study was to assess the health and foot care status of patients with diabetes in Iran. The majority of participants were female (64.8%), married (92.5%), and unemployed (71.1%). Additionally, 96.75 of participants had health insurance, and 88.4% had a high school diploma or lower educational level. The participants’ average age and age range were 57.6 ± 10.21 (mean ± SD) years and 25–88 years. The mean duration of DM was 11.56 ± 7.41 years. Most patients had a family history of DM (73.9%), and about half (54.1%) used only oral hypoglycemic agents. The mean total DFSQ score was 37.92 ± 10.51. The mean of personal self-care, podiatric care, and footwear was also 17.14 ± 5.00, 8.51 ± 3.00, and 12.27 ± 4.45, respectively. As Inlow score of participants according to the ‘Inlow’s 60-Second Screen’ and recommended screening interval suggested in Table 1.

Most patients need to have foot screening for DFU risk stratification annually. For interpreting the results of Inlow’s 60-second, scores of five identified parameters are shown in Table 2. Despite low scores of different parameters, bony changes, and sensation parameters, with higher scores than other parameters, should be considered.

The frequency of look, touch, and assessment indicators related to skin and nail changes in patients showed that more than half had healthy and integrated skin and healthy nails on both feet. However, about one-third of patients had dry skin, mild fungus and calluses, and rough nails (Table 3).

The same analysis showed a statistically significant difference (p ≤ 0.005) in sensation and bony changes parameters scores of Inlow’s 60-second screen according to age, educational status, duration of diabetes, history of DFU, retinopathy, and nephropathy (Table 4).

**Table 1:** Inlow’s 60-Second Screen’ and Recommended Screening Interval (n = 1094)

Foot	N	Left	Right
**Inlow score**	1094		
Mean (SD)		6.04 (4.41)	6.04 (4.38)
Range		0–25	0–25
**Recommended Screening Interval** (Inlow score)		**Left (n)%**	**Right (n)%**
Yearly (0–6)		(641) 58.6%	(645) 59%
Every 6 month (7–12)		(355) 32.4%	(353) 32.2%
Every 3 month (13–19)		(95) 8.7%	(93) 8.5%
1–3 month (20–25)		(3) 0.3%	(3) 0.3%

**Table 2:** Scores of five parameters of Inlow’s 60-Second Screen tool. (n = 1094)

Indications	Indicatives of …	Left	Right	Scores Range
		Mean (SD)		
Self-Care Parameters	Self-care deficit (Q1, Q2, Q4)	1.28 (1.25)	1.28 (1.25)	0–9
Integument Parameters	Integument 1: Callus formation (Q4, Q7)	0.68 (0.79)	0.68 (0.80)	0–7
	Integument 2: Infected ulcer (Q1, Q6, Q12)	0.54 (0.70)	0.54 (0.68)	0–5
	Integument 3: Infected nails (Q2, Q6, Q12)	0.48 (0.66)	0.48 (0.63)	0–4
Arterial Flow Parameters	Peripheral Arterial Disease (Q5, Q10, Q11)	0.28 (0.54)	0.28 (0.55)	0–3
Sensation Parameters	Loss of Protective Sensation / Neuropathy (Q8, Q9)	1.78 (1.97)	1.78 (1.94)	0–6
Bony Changes Parameters	Indicative of Charcot changes (Q3, Q8,9)	2.26 (2.26)	2.28 (2.26)	0–8

**Table 3:** Frequency of foot screening indicators of Inlow’s 60-Second Screen tool. (n = 1094)

**Look – 20 seconds**	Score		
	Left Foot		Right Foot
	N (%)		N (%)
**1 Skin**			
0 = intact and healthy 1 = dry with fungus or light callus 2 = heavy callus build up 3 = open ulceration or history of previous ulcer	657 (60.1) 380 (34.7) 53 (4.8) 4 (0.4)		656 (60) 382 (34.9) 53 (4.8) 3 (0.3)
**2 Nails**			
0 = well-kept 1 = unkempt and ragged 2 = thick, damaged, or infected	705 (64.4) 354 (32.4) 35 (3.2)		702 (64.4) 360 (32.9) 32 (2.9)
**3. Deformity**			
0 = no deformity 2 = mild deformity 4 = major deformity	839 (76.7) 247 (22.6) 8 (0.7)		830 (75.9) 255 (23.3) 9 (0.8)
**4. Footwear**			
0 = appropriate 1 = inappropriate 2 = causing trauma	652 (59.6) 402 (36.7) 40 (3.7)		656 (59.9) 397 (36.3) 42 (3.8)
**Touch – 10 seconds**			
**5. Temperature – Cold**			
0 = foot warm 1 = foot is cold	886 (81.0) 208 (19.0)		876 (80.1) 218 (19.9)
**6. Temperature – Hot**			
0 = foot is warm 1 = foot is hot	1038 (94.9 56 (5.1)		1044 (95.4) 51 (4.6)
**7. Range of Motion**			
0 = full range to hallux 1 = hallux limitus 2 = hallux rigidus 3 = hallux amputation	881 (80.5) 168 (15.4) 42 (3.8) 3 (0.3)		876 (80.1) 179 (16.4) 36 (3.3) 3 (0.2)
**Assess – 30 seconds**			
**8. Sensation – Monofilament Testing**			
0 = 10 sites detected 2 = 7 to 9 sites detected 4 = 0 to 6 sites detected	578 (52.8) 330 (30.2) 186 (17.0)		570 (52.1) 346 (31.6) 178 (16.3)
**9. Sensation – Ask 4 Questions**:			
i. Are your feet ever numb?			
0 = no	822 (75.1)		824 (75.3)
1 = yes	272 (24.9)		270 (24.7)
ii. Do they ever			
0 = no	669 (61.2)		683 (62.4)
1 = yes	425 (38.8)		411 (37.6)
iii. Do they ever			
0 0 = no	619 (56.6)		614 (56.1)
0 = yes	475 (43.4)		480 (43.9)
iv. Do they ever feel like insects are crawling on them?			
0 = no	962 (87.9)		949 (86.7)
1 = yes	132 (12.1)		145 (13.3)
**10. Pedal Pulses**			
0 = present 1 = absent	1007 (92.0) 87 (8.0)		1015 (92.8) 79 (7.2)
**11. Dependent Rubor**			
0 = no 1 = yes	1079 (98.6 15 (1.4)		1071 (97.9) 23 (2.1)
**12. Erythema**			
0 = no 1 = yes	1052 (96.2 42 (3.8)		1055(96.4) 39 (3.6)

**Table 4:** Relationship between the sample characteristics and means of total and parameters scores of Inlow’s 60-Second Screen (n = 1094)

Variables		N (%)	Parameters (Mean ± SD)							
			Self-Care^*^		Integument 1 ^**^	Integument 2 ^**^	Integument 3 ^**^	Arterial Flow^***^	Sensation^****^	Bony Changes^*****^
**Age**	25–50	241 (22.0)	1.14 (1.23)		0.56 (0.66)	0.49 (0.66)	0.33 (0.56)	0.20 (0.44)	1.17 (1.89)	1.43 (2.10)
	51–75	809 (73.9)	1.30 (1.25)		0.69 (0.81)	0.55 (0.69)	0.50 (0.64)	0.31 (0.56)	1.91 (1.92)	2.45 (2.22)
	> 76	44 (4.0)	1.75 (1.31)		1.02 (0.97)	0.61 (0.57)	0.68 (0.60)	0.48 (0.69)	2.73 (1.93)	3.68 (2.36)
	*p-value*		0.010		0.001	0.354	0.000	0.002	0.000	0.000
**Marital status**	Single	36 (3.3)	1.28 (1.44)		0.58 (0.87)	0.56 (0.73)	0.47 (0.73)	0.33 (0.58)	1.33 (2.02)	1.89 (2.57)
	Married	1012 (92.5)	1.26 (1.24)		0.67 (0.79)	0.53 (0.68)	0.47 (0.63)	0.29 (0.55)	1.78 (1.94)	2.27 (2.25)
	Others	Others (4.2)	1.74 (1.35)		0.87 (0.88)	0.72 (0.54)	0.48 (0.50)	0.28 (0.50)	2.00 (1.88)	2.65 (2.31)
	*p-value*		0.042		0.202	0.176	0.994	0.897	0.290	0.313
**Educational Status**	<Diploma	967 (88.4)	1.37 (1.28)		0.76 (0.82)	0.57 (0.70)	0.49 (0.62)	0.34 (0.58)	2.04 (2.00)	2.53 (2.30)
	>Diploma	127 (11.6)	1.12 (1.20)		0.53 (0.72)	0.47 (0.63)	0.42 (0.63)	0.21 (0.47)	1.29 (1.74)	1.80 (2.11)
	*p-value*		0.002		0.000	0.028	0.068	0.000	0.000	0.000
**Type of medications**	Oral	592 (54.1)	1.29 (1.22)		0.66 (0.77)	0.51 (0.64)	0.45 (0.61)	0.25 (0.50)	1.67 (1.87)	2.18 (2.18)
	Insulin	204 (18.6)	1.23 (1.29)		0.68 (0.86)	0.59 (0.73)	0.50 (0.65)	0.30 (0.57)	2.07 (2.19)	2.53 (2.50)
	Both	298 (27.2)	1.30 (1.30)		0.71 (0.79)	0.55 (0.71)	0.49 (0.64)	0.37 (0.60)	1.80 (1.90)	2.29 (2.24)
	*p-value*		0.717		0.616	0.307	0.588	0.011	0.038	0.167
**Family history of diabetes**	Yes	808 (73.9)	1.34 (1.29)		0.73 (0.82)	0.56 (0.69)	0.48 (0.64)	0.29 (0.55)	1.71 (1.93)	2.22 (2.25)
	No	286 (26.1)	1.12 (1.12)		0.52 (0.70)	0.48 (0.64)	0.43 (0.57)	0.30 (0.54)	1.96 (1.98)	2.44 (2.28)
	*p-value*		0.013		0.000	0.102	0.270	0.769	0.068	0.153
**Health insurance**	Yes	1058(96.7)	1.29 (1.26)		0.68 (0.80)	0.54 (0.68)	0.47 (0.63)	0.29 (0.55)	1.80 (1.96)	2.30 (2.27)
	No	36 (3.3)	1.08 (1.15)		0.61 (0.76)	0.36 (0.63)	0.47 (0.65)	0.36 (0.54)	1.22 (1.29)	1.67 (1.75)
	*p-value*		0.337		0.614	0.118	0.975	0.447	0.083	0.101
**Duration of diabetes**	< 15	835 (76.3)	1.26 (1.22)		0.67 (0.79)	0.52 (0.67)	0.47 (0.64)	0.29 (0.54)	1.63 (1.93)	2.11 (2.21)
	> 15	259 (23.7)	1.37 (1.35)		0.69 (0.82)	0.59 (0.69)	0.47 (0.59)	0.31 (0.56)	2.23 (2.06)	2.80 (2.33)
	*p-value*		0.212		0.819	0.111	0.861	0.583	0.000	0.000
**Hypertension**	Yes	640 (58.5)	1.37 (1.29)		0.76 (0.84)	0.56 (0.71)	0.51 (0.66)	0.34 (0.59)	1.94 (1.90)	2.47 (2.23)
	No	454 (41.5)	1.16 (1.19)		0.56 (0.71)	0.50 (0.63)	0.41 (0.58)	0.23 (0.47)	1.55 (1.98)	2.00 (2.27)
	*p-value*		0.009		0.000	0.145	0.012	0.001	0.001	0.001
**CVD**	Yes	324 (29.6)	1.42 (1.32)		0.80 (0.85)	0.55 (0.69)	0.57 (0.68)	0.35 (0.61)	1.91 (1.87)	2.48 (2.20)
	No	770 (70.4)	1.22 (1.22)		0.63 (0.76)	053 (0.67)	0.43 (0.59)	0.27 (0.51)	1.72 (1.98)	2.19 (2.28)
	*p-value*		0.016		0.001	0.666	0.001	0.015	0.151	0.059
**History of DFU**	Yes	115 (10.5)	1.80 (1.46)		1.02 (1.00)	0.81 (0.89)	0.76 (0.82)	0.45 (0.65)	2.75 (2.00)	3.55 (2.48)
	No	979 (89.5)	1.22 (1.21)		0.64 (0.76)	0.50 (0.64)	0.44 (0.59)	0.27 (0.53)	1.66 (1.91)	2.13 (2.18)
	*p-value*		0.000		0.000	0.000	0.000	0.001	0.000	0.000
**Retinopathy**	Yes	283(25.9)	1.43 (1.29)		0.79 (0.88)	0.61 (0.72)	0.57 (0.65)	0.37 (0.60)	2.45 (1.97)	3.00 (2.21)
	No	811 (74.1)	1.23 (1.24)		0.64 (0.76)	0.51 (0.66)	0.43 (0.61)	0.27 (0.52)	1.54 (1.88)	2.02 (2.22)
	*p-value*		0.017		0.005	0.030	0.002	0.008	0.000	0.000
**Nephropathy**	Yes	126 (11.5)	1.48 (1.29)		0.87 (0.95)	0.66 (0.70)	0.57 (0.66)	0.32 (0.58)	2.35 (2.05)	3.20 (2.48)
	No	968 (88.5)	1.26 (1.25)		0.65 (0.77)	0.52 (0.67)	0.46 (0.62)	0.29 (0.54)	1.70 (1.92)	2.18 (2.21)
	*p-value*			0.065	0.005	0.031	0.052	0.582	0.000	0.000

*Self-Care Parameters: Self-care deficit, **Integument Parameters (Integument 1: Callus formation, Integument 2: Infected ulcer, Integument 3: Infected nails), *** Arterial Flow Parameters: Peripheral Arterial Disease, **** Sensation Parameters: Loss of Protective Sensation / Neuropathy, ***** Bony Changes Parameters: Indicative of Charcot changes

## Discussion

In this cross-sectional descriptive study, we evaluated the health and foot care status of 1094 patients with diabetes from 10 provincial capital cities in Iran, which serve as nodes of a diabetic foot network. We utilized the DFSQ and ‘Inlow’s 60-Second Screen’ tools to assess the participants. The current study’s findings showed that personal self-care among Iranian patients with diabetes is better than other dimensions of foot care. Additionally, about half of the diabetic patients who participated in this study across Iran affected by peripheral neuropathy, and more than half of patients should undergo annual foot ulcer screening.

Regarding foot care, the mean total DFSQ score was found to be 37.92 ± 10.51. The mean scores for personal self-care, podiatric care, and footwear were 17.14 ± 5.00, 8.51 ± 3.00, and 12.27 ± 4.45, respectively. Personal self-care emerged as the best dimension. In line with these results, another study conducted in Tehran, the capital of Iran, particularly focusing on women with type 2 diabetes, identified foot self-care as the most important dimension of DFSQ. The scores for personal care, podiatric care, and footwear in that study were reported as 16.98 ± 7, 5.95 ± 2.11, and 12.26 ± 3.95, respectively. Although the Tehran study only evaluated the population of one province in Iran, its findings regarding foot care dimensions align closely with our current research [21]. However, the average total DFSQ score of 60.38 ± 9.9 among diabetic women in Tehran was higher than the current study. The economic characteristics of the study population would be an essential factor to consider, as another study conducted in Iran found that self-care behaviors in low-income patients with diabetes were not appropriate [26].

The current study’s findings showed that the mean total DFSQ score was 37.92 ± 10.51. The mean score of personal self-care, podiatric care, and footwear was also 17.14 ± 5.00, 8.51 ± 3.00, and 12.27 ± 4.45. Personal self-care is considered the best dimension. In line with these results, another study in Tehran, the capital of Iran, especially in women with type 2 diabetes, suggested foot self-care as the best dimension of DFSQ. Additionally, the personal care, podiatric care, and footwear scores reported 16.98 ± 7, 5.95 ± 2.11, and 12.26 ± 3.95, respectively. Although the study evaluated the population of Tehran as just a province of Iran’s population, their results of foot care dimensions are mainly consistent with the findings of current research [21]. However, the average total DFSQ score of 60.38 ± 9.9 among diabetic women in Tehran was higher than the present study. The economic characteristics of the study population would be an essential factor to consider, as another study conducted in Iran found that self-care behaviors in low-income patients with diabetes were inadequate [26].

The results of Inlow’s 60-Second Screen showed that about 59% of the patients have an annual visit for examination due to a low risk of DFU (with a score of 0 to 6). This indicates that more than 40% of patients with diabetes need foot examination at intervals of less than one year, and less than 0.5% should refer for examination between 1 and 3 months. Another study conducted in the south of Iran, following the guidelines of the International Working Group on the Diabetic Foot (IWGDF), recommended different follow-up intervals based on risk levels: group 0 annually, group 1 every 3 to 6 months, group 2 every 2 to 3 months, and group 3 every 1 to 2 months [27]. Similarly Moradi et al. review study on type 2 diabetic patients in Iran, suggested annual check-ups in general and less than 3-month check-ups for patients at higher risk of foot ulcers [4]. Furthermore, O’Brien et al. study focusing on educational interventions for diabetic patients found that regular examinations every six months for diabetic patients and every three months for high-risk individuals can significantly improve the prevalence of foot ulcers in patients with diabetes [28].

Based on the results of the evaluation of the indicators defined in Inlow’s 60-Second Screen of this study, it was determined that the patients in 3 parameters of this indication, including bone changes, sensory status, and self-care, had mean scores of 2.28, 1.78 and 1.28, respectively. This showed that patients with bony changes and loss of protective sensation due to insufficient self-care could be prone to neuropathic foot ulcers. A similar study conducted in Ahvaz indicated the presence of deformity in one-fifth of the participating patient’s [27]. In line with the results obtained, the findings of a systematic review and meta-analysis conducted in the Iranian population indicated that the prevalence of diabetic neuropathy varies from 16 to 87%, and the total prevalence of peripheral neuropathy is estimated to be 53% using random effect.

This study showed that the prevalence of peripheral neuropathy among individuals with diabetes in Iran seems to be very high. More than half of the patients with diabetes have diabetic neuropathy [29]. In a study conducted by Campbell et al., a prevalence of approximately 27% of sensory disorders was reported, with a focus on neuropathy in the UK population. Therefore, it is recommended that in addition to continuous patient education, foot examination in short intervals be conducted to prevent neuropathic wounds [5]. In a study conducted on adult diabetic patients in the diabetes clinic at Gondar University Hospital in northwestern Ethiopia, researchers found that diabetic patients with neuropathy were 21.7 times more likely to develop diabetic foot ulcers than patients with diabetes without neuropathy. This increased risk can be attributed to microvascular complications and neuropathy in diabetic patients with elevated blood glucose levels. Neuropathy can contribute to foot ulcers due to heightened pressure load. Additionally, patients with diabetes who neglected foot care were found to be 2.5 times more susceptible to DFU than their counterparts. Implementing proper self-care practices, such as regular foot washing, thorough drying, daily assessment of foot condition, promoting blood circulation, and proactive management of any abnormalities, can effectively prevent the occurrence of diabetic foot ulcers [30].

Additionally, screening using Inlow’s 60-Second Screen in four areas of the skin and nails, the frequency of changes, LOPS peripheral neuropathy, PAD peripheral vascular disease, and foot deformity, and the condition of the foot covering showed that more than 30% of patients suffer from dry skin with mild fungus, calluses as well as rough and uneven nails. These patients have mild bone deformities (about 23%) and have inappropriate foot covering (about 36%). Based on the monofilament test, it was determined that about half of the patients (about 47%) are at risk of neuropathy, and the scores related to the four sensory questions of Inlow’s 60-Second Screen also confirm this finding. This group of patients is susceptible to neuropathy DFU, which could prevent with appropriate preventive interventions. Accordingly, the physical examination of the skin in patients with diabetes in one southern city in Iran showed that dry skin is the most common skin disorder, with a frequency of 19% among diabetics [27]. Pavicic et al., in their study on diabetic patients, found that skin lesions with fungus and calluses in patients with neuropathy often lead to infections. Factors include improper footwear, improper foot care, or neglect of foreign objects, often accompanied by structural deformation of the foot caused by DFUs. Therefore, rapid neurological and vascular diagnostic studies, regular foot examinations, and primary preventive measures play an essential role in preventing and detecting foot ulcers. Acquiring the treatment goals as optimal control of diabetes, relief of pressure points, and prevention or reduction of callus formation would be considered [31].

The findings of this study indicate that individuals with cardiovascular diseases should pay more attention to their footwear choices and foot care practices. In a study examining a team approach to managing diabetic foot, Sumpio et al. discovered that peripheral neuropathy, a significant complication of diabetes, can disrupt tissue arterial blood flow and lead to the development of wounds. Untreated inadequate limb perfusion may result in incurable wounds and necessitate amputation. Therefore, diabetic patients with the risk of CVD should pay more attention to the proper care of their feet and reduce the incidence of complications as much as possible by observing hygiene and regular examinations [32]. In line with the current research, Hingorani et al. also reported in their study that diabetes is a risk factor for patients with cardiovascular disorders, and the combination of these two diseases increases the probability of developing wound and subsequent amputation by about 50%. These researchers found that diabetic patients with vascular disorders need more attention to the health of their feet than others because they believe that having sufficient knowledge about proper care, washing, and regular examination of the feet, along with the control of cardiovascular disease significantly mitigate the hazardous consequences of the disease [33].

On the other hand, the educational level of the people studied in this research, except for the self-care item, had an inverse and significant correlation with Inlow’s 60-Second Screen parameters, which indicates that with the increase in the education level of the person, bone deformation, sensory disorders, and skin and nail problems decrease significantly and self-care increases. Along with the findings of this research, other studies also emphasize the importance of the level of education and the level of knowledge of diabetic patients in preventing the occurrence of complications in patients with diabetes. Although studies have been done before regarding foot care in Iranian patients with diabetes, indicating poor awareness and subsequent inappropriate performance of diabetic Iranians [21, 34], it is clear that educational progress has a significant correlation with health behaviors and significantly improves self-care in people with diabetes [35].

The present research findings reveal a significant association between the duration of diabetes and the incidence of sensory disorders and foot deformities. This study highlights that individuals with a history of diabetes for more than 15 years are more prone to bone deformities and sensory disorders than others. In this regard, in the study of Booya et al., a significant relationship was reported between the duration of the disease and the incidence of risk factors in the diabetes [34]. Allan et al., in their review study, stated that the incidence of bone deformity has a significant relationship with increasing age, especially in people with a prolonged history of diabetes [36].

An investigation into the relationship between indicators such as a history of high blood pressure, cardiovascular diseases, diabetic ulcers, retinopathy, and nephropathy with the parameters examined in this study using Inlow’s 60-Second Screen revealed significant associations with sensory disorders, skin issues, and nail problems. The current research findings demonstrate that individuals with a history of diabetic ulcers and neuropathy exhibit increased measurements of all parameters assessed by Inlow’s 60-Second Screen, indicating a higher risk for diabetic foot ulcers (DFUs). Consequently, individuals who have previously experienced diabetic ulcers and those with a history of diabetes-related retinopathy should undergo more frequent examinations due to their susceptibility to diabetes complications such as DFUs. The results of other studies conducted in this field also confirm the findings of the present study [37, 38]. Thus, it can be concluded that the presence of any diabetes complications, including cardiovascular diseases, retinopathy, and nephropathy, in diabetic patients increases the likelihood of developing foot ulcers [39, 40].

## Conclusion

The results of the present study indicate that due to complications such as dry skin with fungus and calluses, mild bone deformities, and improper footwear, about half of the patients are at risk of neuropathy foot ulcer. Notably, the podiatric care status was the lowest care dimension among patients with diabetes. The presence of peripheral neuropathy and a lack of podiatric care would be considered the leading risk factor for developing DFU in patients with diabetes in an Iranian population study. Accordingly, providing continuous patient education on personal self-care, proper footwear, and foot examination at regular intervals based on recommended screening interval of Inlow score should be considered for DFU prevention and early diagnosis of ulcers and timely therapeutic interventions.

### Limitations

There were some limitations in this study; first, the number and sampling of participants were raised from 10 cities in Iran, which covered only some of the diversity of the Iranian population since Iran is a vast country with different races, ethnicities, and cultural habits. Second, there was no further follow-up in this cross-sectional study. Despite the mentioned limitations, it would be noted that limited examinations have been conducted regarding diabetic foot self-care and foot screening at risk for developing DFUs in the Iranian population, which the current study has considered.

## Data Availability

Data is available on request from the authors. If someone wants to request the data from this study should be contacted Maryam Aalaa.

## Abbreviations

DM: Diabetes Mellitus
DFU: Diabetic Foot Ulcer
